# Scalable secretory production of influenza A (H1N1) hemagglutinin HA1 in *Pichia pastoris* through expression and process optimization

**DOI:** 10.1186/s12934-026-02985-0

**Published:** 2026-03-17

**Authors:** Zeliang Guo, Wenting Ding, Chendi Yang, Xiaoying Zhi, Yu Zhang, Fengjia Xi, Rongzeng Liu

**Affiliations:** 1 Department of Immunology, College of Basic Medicine and Forensic Medicine, Henan University of Science and Technology, Luoyang, China; 2 Key Laboratory of Clinical Immunology and Inflammatory Diseases, Luoyang, China

**Keywords:** *Pichia pastoris*, recombinant protein expression, hemagglutinin HA1, secretory expression, microbial cell factory, viral glycoprotein

## Abstract

**Background:**

Efficient and scalable production of viral antigens remains a key challenge in the development of recombinant subunit vaccines and diagnostic reagents. Microbial expression systems, particularly *Pichia pastoris* (*P. pastoris*), offer a promising platform for producing complex viral glycoproteins with appropriate folding and post-translational modifications.

**Results:**

In this study, the hemagglutinin head domain (HA1) of influenza A (H1N1) virus was expressed as a secreted recombinant protein in *P. pastoris* GS115. The HA1 gene was codon-optimized and expressed under the control of the methanol-inducible AOX1 promoter with an α-factor signal peptide. Multicopy integrants were enriched using G418 selection, and expression conditions were systematically optimized. Under shake-flask induction, the selected recombinant strain produced up to 0.375 g/L of rHA1 in the culture supernatant. The protein was efficiently purified by Ni-NTA affinity chromatography to a purity exceeding 95%. PNGase F digestion confirmed N-linked glycosylation. Limited functional validation demonstrated that the yeast-expressed rHA1 retained antigenic integrity, as evidenced by the induction of rHA1-specific antibodies and hemagglutination-inhibiting activity in a murine model.

**Conclusions:**

These results establish *P. pastoris* as an effective microbial cell factory for the high-level secretion of influenza HA1 protein. The optimized expression and purification strategy provides a scalable and cost-efficient framework for microbial production of viral antigens and may be applicable to other glycoproteins of biomedical relevance.

## Introduction

Influenza A virus subtype H1N1 remains a continuously evolving viral pathogen, primarily driven by antigenic drift in its surface glycoproteins. This genetic variability complicates long-term antigen stability and poses ongoing challenges for the preparation of well-characterized viral antigens for immunological and virological research [1–3]. Among the viral surface proteins, hemagglutinin (HA) plays a central role in viral attachment and entry into host cells and represents a key antigenic determinant of influenza A viruses.

Structurally, HA is synthesized as a precursor that is cleaved into two subunits, HA1 and HA2. The HA1 subunit forms the globular head region and contains the receptor-binding domain (RBD) as well as multiple antigenic sites recognized by host antibodies [4–7]. Although the HA1 region undergoes frequent antigenic variation, structural and immunological studies have identified conserved elements within the RBD that are critical for receptor engagement and antibody recognition. These features make the HA1 head domain a valuable antigen for studying virus-host interactions and antigen-specific immune responses [8–10].

The availability of recombinant HA1 with appropriate folding, solubility, and antigenic integrity is essential for such studies; however, efficient production of this domain remains technically challenging. Conventional expression systems, including *Escherichia coli* (*E. coli*) and mammalian cell platforms, each present inherent limitation. While *E. coli* enables rapid and cost-effective protein production, it lacks the capacity to perform post-translational modifications such as glycosylation, which are important for the structural stability and antigenic properties of HA [11–13]. Mammalian expression systems, although capable of producing properly glycosylated HA proteins, are often constrained by high production costs, longer development timelines, and limited scalability [14, 15].

In this context, the methylotrophic yeast *Pichia pastoris* (*P. pastoris*) has emerged as an attractive alternative host for recombinant protein production. This system combines rapid growth, genetic stability, and efficient secretion under high cell density cultivation with the ability to perform eukaryotic protein folding and glycosylation. Expression driven by the alcohol oxidase 1 (AOX1) promoter allows for strong, tightly regulated induction, making *P. pastoris* particularly suitable for the production of complex recombinant proteins [16–19]. Despite these advantages, challenges remain, including variable gene integration efficiency, dependence on methanol induction, and suboptimal expression of certain heterologous proteins due to codon bias or limitations in folding and secretion pathways [20–22].

To address these challenges, multiple strategies have been developed to enhance recombinant protein yields in *P. pastoris*, such as selection of multi-copy integrants, codon optimization, and systematic optimization of induction parameters. When appropriately combined, these approaches can significantly improve expression levels and facilitate the production of soluble, functionally relevant proteins.

In the present study, we focused on the recombinant production of the HA head domain (HA1) from Influenza A (H1N1) virus using the *P. pastoris* GS115 expression system. By integrating multi-copy screening, codon optimization, and process optimization, we achieved efficient secretion of soluble recombinant HA1 (rHA1). The expression characteristics, purification, and basic immunological properties of the resulting rHA1 protein were systematically evaluated. This work establishes an optimized strategy for producing influenza virus HA1 antigen in *P. pastoris* and provides a practical basis for generating recombinant viral antigens for downstream immunological and virological applications.

## Materials and methods

### Construction of recombinant expression vector

**Fig. 1:**
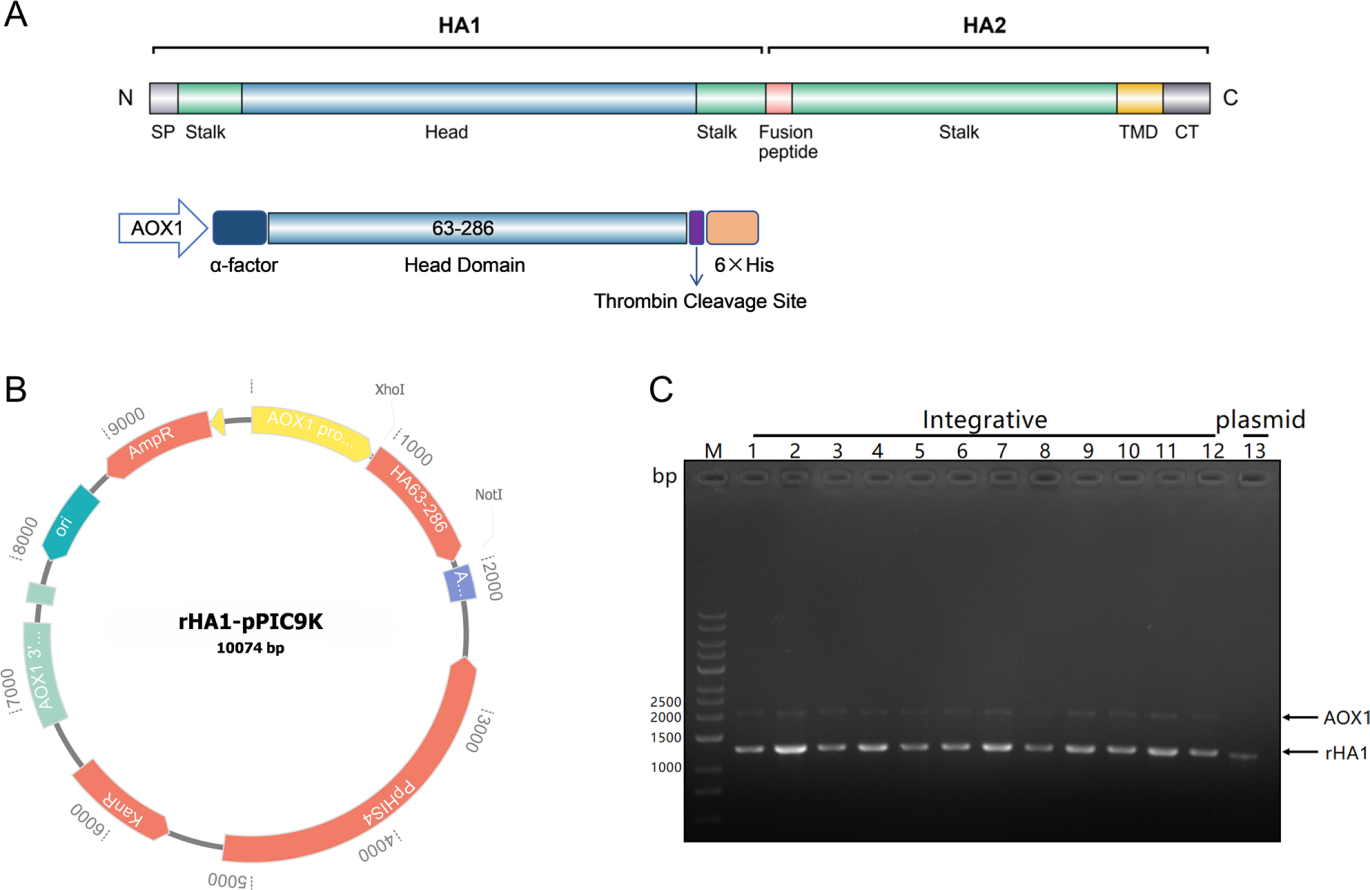
Construction of the recombinant expression plasmid and confirmation of genomic integration of the target gene. **A** Schematic representation of the HA1 region derived from influenza A (H1N1) hemagglutinin. **B** Schematic map of the recombinant secretory expression plasmid rHA1-pPIC9K under control of the AOX1 promoter. **C** PCR analysis confirming genomic integration of the rHA1 expression cassette at the AOX1 locus in *P. pastoris*. Lane M, DNA molecular weight marker; lanes 1–12, representative multicopy transformants showing both the endogenous AOX1 (~ 2.2 kb) and the integrated rHA1 cassette (~ 1.29 kb); lane 13, rHA1-pPIC9K plasmid used as a positive control, showing only the rHA1 cassette (~ 1.29 kb)

The HA1 coding sequence (amino acids 63–286) derived from influenza A virus strain A/California/07/2009 (GenBank accession number CY121680.1) was codon-optimized for expression in *P. pastoris* and synthesized commercially (GenScript, China). For secretory expression, the optimized HA1 gene was cloned in-frame downstream of the *P. pastoris* α-factor signal peptide in the pPIC9K vector, which is driven by the methanol-inducible AOX1 promoter. A C-terminal hexahistidine (6×His) tag was introduced to facilitate affinity purification, with a thrombin cleavage site inserted between the HA1 sequence and the His-tag to allow optional tag removal. The resulting recombinant plasmid was designated rHA1-pPIC9K (Fig. 1B). The codon-optimized HA1 gene was inserted into the pPIC9K vector at the *Xho I* and *Not I* restriction sites downstream of the AOX1 promoter to generate the recombinant expression construct.

### Plasmid amplification and transformation of *P. pastoris*

The recombinant plasmid rHA1-pPIC9K was propagated in *E. coli* DH5α cells and amplified by cultivation in Luria-Bertani (LB) medium supplemented with ampicillin at 37 °C for 12 h. Plasmid DNA was isolated using a commercial miniprep kit (Sangon Biotech, China). For genomic integration, the recombinant plasmid was linearized with *SalI* restriction enzyme (New England Biolabs, USA), purified, and quantified using a NanoDrop One spectrophotometer (Thermo Fisher Scientific, USA). Linearization was verified by agarose gel electrophoresis. Electrocompetent *P. pastoris* GS115 cells (Beyotime Biotechnology, China) were transformed with the linearized plasmid by electroporation (25 µF, 200 Ω, 2.5 kV; Bio-Rad MicroPulser). Transformants were selected on histidine-deficient minimal dextrose (MD) agar plates and incubated at 30 °C for 3 days to obtain His⁺ colonies.

### Selection of multicopy integrants and phenotype identification

The pPIC9K vector contains a kanamycin resistance gene that confers resistance to geneticin (G418) in *P. pastoris*, enabling selection of multicopy integrants. To enrich for clones harboring multicopy integrants, His⁺ transformants were subjected to stepwise selection on YPD agar plates containing increasing concentrations of G418 (2.0 and 4.0 mg/mL). Genomic DNA was prepared from selected transformants using a rapid boiling-freezing-boiling method as previously described [23]. Integration of the rHA1 expression cassette was confirmed by PCR using AOX1-specific primers. Methanol utilization phenotypes were determined by assessing growth on minimal methanol (MM) and minimal dextrose (MD) agar plates. Transformants exhibiting robust growth on both media were classified as Mut⁺. Confirmed strains were cultured in BMGY medium to an OD_600_ of 2–6, mixed with sterile glycerol (final concentration 40%, v/v), and stored at − 80 °C.

### Small-scale expression screening by methanol induction

Recombinant *P. pastoris* strains were cultivated in 10 mL BMGY (1% yeast extract, 2% peptone, 100 mM phosphate buffer pH 6.0, 1.34% Yeast nitrogen base, 0.02% Biotin, 1% glycerol) medium at 30 °C with agitation at 250 rpm until reaching an OD_600_ of between 2 and 6. Cells were harvested by centrifugation and resuspended in 10 mL BMMY induction medium. Methanol was added every 24 h to a final concentration of 0.5% (v/v) to maintain induction of the AOX1 promoter. Samples were collected at 0, 24, 48, 72, and 96 h post-induction. Expression levels of rHA1 in the culture supernatants were analyzed by SDS-PAGE, and protein identity was confirmed by Western blotting using an anti-His tag antibody.

### Scale-up cultivation and purification of rHA1

For large-scale production, the high-expression clone rHA1-E4 was cultivated in 100 mL BMGY medium in a 500 mL baffled flask at 30 °C and 250 rpm until the OD_600_ reached 2–6. Cells were collected by centrifugation (3000 g, 5 min) and resuspended in 500 mL BMMY induction medium in a 2 L baffled flask to an initial OD_600_ of approximately 1.0, as described previously [24]. Induction was carried out at 30 °C for 96 h with methanol supplementation (0.5%, v/v) every 24 h. All expression experiments were performed in triplicate, and representative data are shown.

After induction, cultures were centrifuged at 4000 g for 30 min at 4 °C, and the clarified supernatant was collected for purification. Recombinant HA1 was purified using Ni-NTA affinity chromatography on an ÄKTA Pure system (GE Healthcare). After washing, bound protein was eluted with imidazole-containing buffer. Protein concentration was determined using a bicinchoninic acid (BCA) assay, and buffer exchange into phosphate-buffered saline (PBS) was performed using centrifugal ultrafiltration devices. Protein purity was assessed by SDS-PAGE followed by densitometric analysis. Glycosylation of rHA1 was evaluated by treatment with peptide-N-glycosidase F (PNGase F) according to the manufacturer’s instructions, with untreated samples serving as controls.

### Functional characterization of rHA1

The following experiments were performed to assess the antigenicity and functional integrity of the recombinant HA1 protein produced in *P. pastoris*.

#### Animal immunizations

Specific-pathogen-free male BALB/c mice (6–8 weeks old) were housed under controlled environmental conditions and handled in accordance with institutional ethical guidelines approved by Henan University of Science and Technology. Mice were randomly divided into three groups: adjuvant control, low-dose, and high-dose immunization groups (*n* = 10 per group). Recombinant HA1 protein was emulsified with complete Freund’s adjuvant (primary immunization) or incomplete Freund’s adjuvant (boosters) at a 1:1 (v/v) ratio. Mice were immunized subcutaneously with either 5–20 µg of rHA1 per dose. Booster immunizations were administered at 14-day intervals for a total of three boosts. Serum samples were collected prior to each immunization and stored at − 80 °C.

#### Detection of rHA1-specific antibody responses

Indirect ELISA was performed to determine rHA1-specific IgG levels in mouse sera. Microtiter plates were coated overnight at 4 °C with purified rHA1 protein (1 µg/mL). After blocking, serially diluted sera were incubated in the plates, followed by incubation with HRP-conjugated goat anti-mouse IgG. Color development was achieved using TMB substrate, and absorbance was measured at 450 nm.

#### Hemagglutinin inhibition assay

Hemagglutination inhibition (HI) assays were performed according to World Health Organization guidelines [25]. Sera were treated with receptor-destroying enzyme prior to testing. HI titers were determined using influenza A (H1N1) HA antigen (A/California/07/2009) and 1% guinea pig red blood cells. The HI titer was defined as the reciprocal of the highest serum dilution that completely inhibited hemagglutination. The HI assay was included to confirm the functional relevance of the recombinant HA1 protein expressed in yeast.

#### Cellular response analysis

Total RNA was extracted from mouse spleens and reverse-transcribed into cDNA. Quantitative PCR was performed using SYBR Green chemistry to assess the expression of IFN-γ, IL-4, T-bet, and GATA-3. Relative gene expression was calculated using the 2^−ΔΔCt^ method with β-actin as the internal reference. Analysis of cytokine- and transcription factor-associated gene expression was limited to evaluating immune response polarization as an indicator of protein functionality, without further mechanistic interpretation.

Splenic lymphocytes were isolated by density gradient centrifugation and cultured in RPMI 1640 medium supplemented with 10% fetal bovine serum. Cells were stimulated with rHA1 protein, concanavalin A, or medium alone. Cell proliferation was assessed using the CCK-8 assay, and absorbance was measured at 450 nm. This assay served as supplementary evidence supporting the retained biological activity of the recombinant rHA1 protein.

### Statistical analysis

Data were analyzed using GraphPad Prism version 9. Results are presented as mean ± standard deviation (SD). Statistical significance was evaluated using one-way or two-way ANOVA, or the Kruskal-Wallis test where appropriate. A value of *p* < 0.05 was considered statistically significant.

## Results

### Transformation of *P. pastoris* GS115 and selection of multi-copy integrants

A schematic representation of the H1N1 HA head domain within the context of the full-length HA protein and the corresponding secretory expression construct is shown in Fig. 1. Following construction of the recombinant plasmid rHA1-pPIC9K, the expression cassette was introduced into *P. pastoris* GS115 for genomic integration and recombinant strain generation. The linearized rHA1-pPIC9K plasmid was introduced into *P. pastoris* GS115 by electroporation. Transformants were initially selected on MD plates, yielding a total of 372 His⁺ colonies. To enrich for strains with potentially higher gene copy numbers, transformants were further screened using increasing concentrations of geneticin (G418). Twenty-five colonies exhibited resistance at 2.0 mg/mL G418, and two colonies (designated C11 and E4) remained viable at 4.0 mg/mL, suggesting the presence of multiple integrated copies of the expression cassette.

Genomic integration of rHA1 at the AOX1 locus was confirmed by PCR analysis using primers flanking the AOX1 promoter region. Representative transformants consistently produced two amplicons: an approximately 2.2 kb fragment corresponding to the endogenous AOX1 gene and a second fragment of 1.29 kb corresponding to the integrated rHA1 expression cassette (Fig. 1C). As expected, the rHA1-pPIC9K plasmid used as a positive control (lane 13) yielded only the 1.29 kb fragment, as it contains the rHA1 cassette but lacks *P. pastoris* genomic DNA. Phenotypic analysis further demonstrated that all tested transformants exhibited a Mut⁺ phenotype, as evidenced by growth on both minimal methanol (MM) and MD plates, indicating retention of a functional AOX1 promoter. Collectively, these results confirmed stable genomic integration of the rHA1 expression cassette in *P. pastoris* GS115.

### Expression and optimization of rHA1 production in *P. pastoris*

**Fig. 2:**
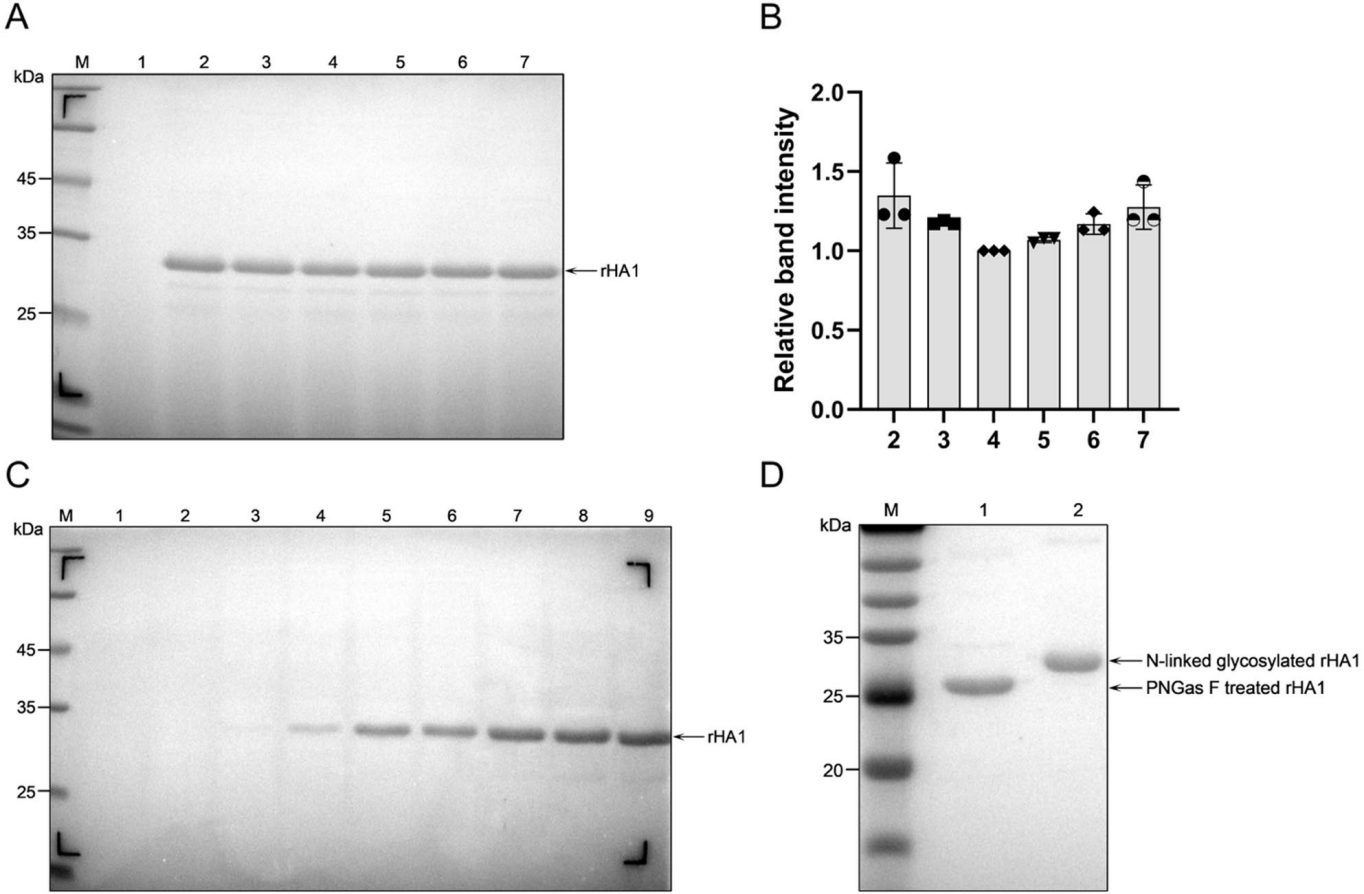
Screening, optimization, and scale-up expression of rHA1 in *P. pastoris*. **A** SDS-PAGE analysis of rHA1 expression during small-scale methanol induction. Lane M, protein molecular weight marker; lane 1, pre-induction control; lanes 2–7, representative recombinant strains (E4, C11, A5, B12, H11, and E12). **B** Densitometric analysis of rHA1 expression levels corresponding to panel (A). **C** SDS-PAGE analysis of rHA1-E4 expression during scale-up cultivation under optimized induction conditions. Lane M contains the protein molecular weight marker; Lanes 1 and 2 represent pre-induction controls (0 h); Lanes 3 and 4 correspond to rHA1-E4 samples cultured for 24 h; Lanes 5 and 6 for 48 h; Lanes 7 and 8 for 72 h; and Lane 9 for 96 h, all under optimized cultivation conditions. **D** SDS-PAGE analysis of purified rHA1 before and after PNGase F treatment, demonstrating N-linked glycosylation. Lane M, protein molecular weight marker; lane 1, PNGase F-treated rHA1; lane 2, untreated rHA1

Putative multi-copy transformants were subjected to small-scale methanol induction in 10 mL cultures to evaluate recombinant protein expression. SDS-PAGE analysis of culture supernatants revealed a distinct protein band at approximately 30 kDa in several strains following methanol induction, consistent with the predicted molecular weight of rHA1 (Fig. 2A, B). Western blot analysis using an anti-His antibody confirmed the identity of the expressed protein and indicated specific expression without detectable non-specific bands.

Among the strains tested, clone rHA1-E4 consistently exhibited the highest expression level and was therefore selected for subsequent optimization experiments. Key induction parameters, including methanol concentration, initial cell density, and induction duration, were systematically evaluated. Optimal expression was achieved using 0.5% methanol, an initial OD_600_ of 1.0, and a 96-h induction period.

Under these optimized conditions, rHA1-E4 was scaled up to a 500 mL induction culture. SDS-PAGE analysis demonstrated that rHA1 secretion became detectable as early as 24 h post-induction and remained stable throughout the induction period (24–96 h), consistently appearing as a single band at approximately 30 kDa (Fig. 2C). Quantitative analysis of the culture supernatant at 96 h indicated that the extracellular rHA1 concentration reached approximately 0.375 g/L, demonstrating efficient secretion and scalability of the optimized expression process.

### Purification and biochemical characterization of rHA1

**Fig. 3:**
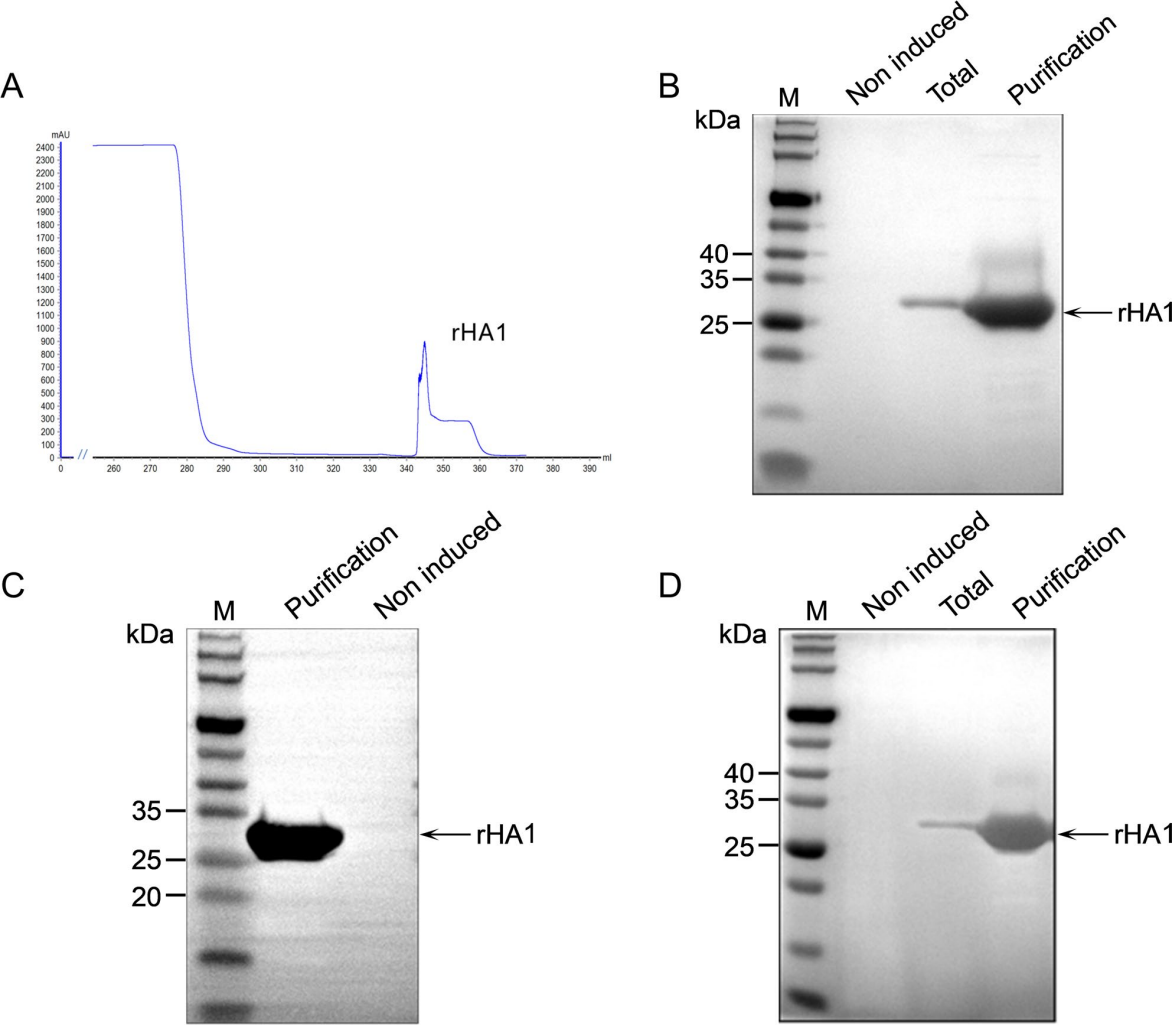
Purification and biochemical characterization of secreted rHA1. **A** Elution profile of rHA1 obtained by Ni-NTA affinity chromatography, showing a single symmetrical peak. **B** SDS-PAGE analysis of purification steps. Lane 1, protein molecular weight marker; lane 2, pre-induction control; lane 3, culture supernatant; lane 4, purified rHA1 following affinity chromatography. **C** Western blot analysis confirming the identity of purified rHA1 using an anti-His antibody. Lane 1, protein molecular weight marker; lane 2, purified rHA1; lane 3, pre-induction control. **D** SDS-PAGE analysis of rHA1 following desalting and buffer exchange. Lane 1, protein molecular weight marker; Lane 2, pre-induction control; Lane 3, culture supernatant; and Lane 4, the rHA1 protein sample after both affinity chromatography and desalting treatment

Culture supernatants harvested after 96 h of induction were subjected to Ni-NTA affinity chromatography for purification of rHA1. Chromatographic analysis revealed a single, symmetrical elution peak with minimal background signal, indicating efficient enrichment of the target protein (Fig. 3A). SDS-PAGE analysis of the eluted fractions showed a predominant band at approximately 30 kDa with minimal detectable contaminants (Fig. 3B). Western blot analysis using an anti-His monoclonal antibody further confirmed the identity of the purified rHA1 protein (Fig. 3C).

Following desalting and buffer exchange, SDS-PAGE analysis demonstrated retention of protein integrity with reduced background staining (Fig. 3D). Protein concentration, determined by BCA assay, corresponded to a concentrated rHA1 preparation suitable for downstream analyses. Densitometric analysis of SDS-PAGE gels using ImageJ indicated that rHA1 accounted for 96.22% of the total protein content in the purified preparation.

To assess post-translational modification, purified rHA1 was treated with PNGase F. Enzymatic deglycosylation resulted in a clear shift in electrophoretic mobility compared to the untreated protein, consistent with the removal of N-linked glycans (Fig. 2D). These results confirm that rHA1 expressed in *P. pastoris* undergoes N-linked glycosylation, consistent with eukaryotic processing.

### Antigen-specific antibody responses following rHA1 administration in mice

**Fig. 4:**
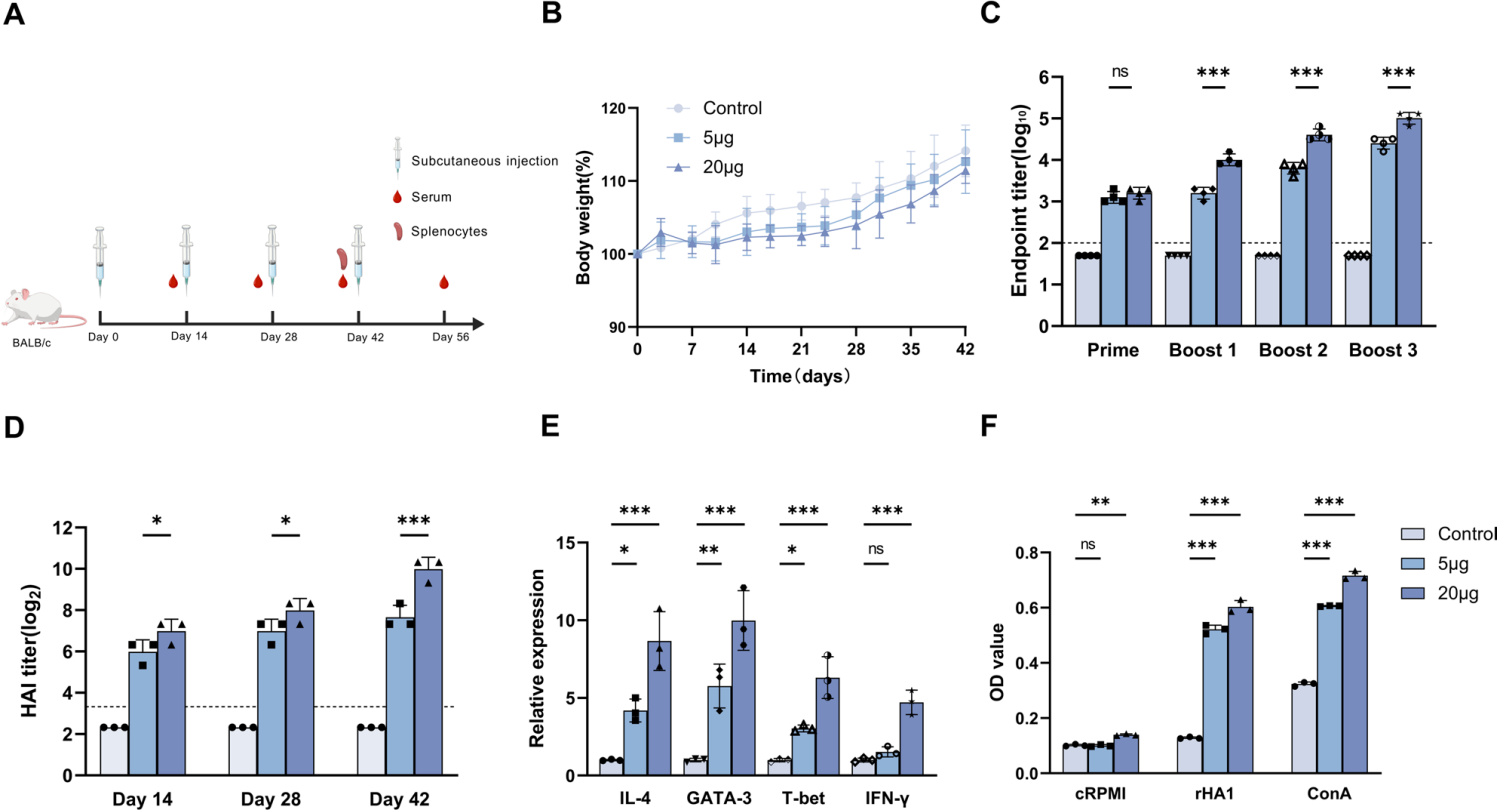
Functional characterization of yeast-derived rHA1 in a murine model. **A** Schematic overview of the experimental schedule for in vivo evaluation and sample collection. **B** Monitoring of body weight changes in mice throughout the experimental period, indicating general tolerability of the recombinant protein. **C** Detection of rHA1-specific IgG responses in mouse sera by indirect ELISA following administration of recombinant protein formulations. Dashed line indicates the assay detection threshold. **D** HI activity of sera against influenza A H1N1 HA (A/California/07/2009). HI titers are expressed as log2-transformed values; dashed line indicates the detection limit. **E** Relative mRNA expression levels of selected cytokines and transcription factors in splenocytes, quantified by RT-qPCR and normalized to β-actin. **F** Antigen-stimulated splenocyte proliferation measured by CCK-8 assay. Statistical significance is indicated as ns, not significant; *p < 0.05; **p < 0.01; ***p < 0.001

To evaluate the antigenic properties of the recombinant protein, mice were immunized with purified rHA1, and serum samples were collected at defined time points (Fig. 4A). No significant differences in body weight were observed between rHA1-treated and control groups throughout the experimental period, indicating good tolerability (Fig. 4B).

Serum levels of rHA1-specific IgG were quantified by indirect ELISA (Fig. 4C). Mice receiving 5 µg of rHA1 exhibited endpoint IgG titers of 1:32,000 following the final immunization, whereas mice administered 20 µg of rHA1 developed significantly higher titers of up to 1:128,000. No antigen-specific IgG responses were detected in the PBS-treated control group within the assay detection limits.

### Functional characterization of rHA1-induced antibodies

The functional activity of antibodies elicited by rHA1 was evaluated using an HI assay with Influenza A (H1N1) antigen (A/California/07/2009). Sera from mice immunized with 5 µg of rHA1 displayed HI titers of 1:320, whereas sera from the 20 µg group exhibited significantly higher HI titers of up to 1:1280 (Fig. 4D). No HI activity was detected in sera from PBS-treated control mice. These results indicate that antibodies induced by yeast-expressed rHA1 retain functional HA-binding activity.

### Cellular immune response indicators following rHA1 exposure

To further characterize immune responses associated with rHA1 administration, the expression levels of Th1- and Th2-associated cytokines and transcription factors were analyzed by RT-qPCR. Mice receiving the high-dose rHA1 formulation exhibited significant upregulation of IFN-γ, IL-4, T-bet, and GATA-3 transcripts compared with the control group (*p* < 0.001), indicating activation of both Th1- and Th2-associated pathways (Fig. 4E). In the low-dose group, significant increases were observed for GATA-3 (*p* < 0.01) and T-bet (*p* < 0.05), whereas IFN-γ expression did not differ significantly from the control group.

Lymphocyte proliferation assays further demonstrated that splenocytes from rHA1-immunized mice exhibited significantly enhanced proliferative responses upon in vitro stimulation with recombinant HA protein compared with PBS controls (*p* < 0.001) (Fig. 4F). No significant differences were observed among groups cultured in medium alone, confirming antigen-specific cellular responsiveness.

## Discussion

The continuous antigenic drift of influenza A viruses necessitates the development of flexible and scalable expression platforms capable of rapidly producing antigenically relevant viral proteins [26, 27]. In this study, we established a *P. pastoris*-based secretory expression system for the production of the HA1 head domain of influenza A (H1N1) virus and systematically evaluated its expression performance, purification efficiency, and basic functional integrity. The primary objective of this work was to develop and optimize a microbial production strategy for recombinant HA1.

The HA1 subunit contains the receptor-binding domain and multiple immunodominant epitopes, making it a structurally complex glycoprotein that presents challenges for heterologous expression [28–30]. Prokaryotic expression systems such as *E. coli* frequently yield misfolded or insoluble HA proteins requiring extensive refolding, whereas mammalian cell systems, although capable of producing functional HA, are constrained by high costs and limited scalability [31, 32]. Yeast expression platforms, particularly *P. pastoris*, offer a balance between these systems by supporting eukaryotic post-translational modifications while retaining the advantages of microbial growth and scalability [33, 34]. Consistent with previous reports, our results demonstrate that *P. pastoris* efficiently secretes soluble HA1 protein with N-linked glycosylation, supporting its utility as a microbial cell factory for viral antigen production.

To enhance recombinant protein yield, multiple optimization strategies were employed, including codon optimization, AOX1 promoter-driven expression, α-factor–mediated secretion, and enrichment of multicopy integrants using G418 selection. Codon optimization has been widely reported to improve translational efficiency and mRNA stability in *P. pastoris*, resulting in enhanced recombinant protein yields [35, 36]. Similarly, AOX1-based expression systems are known for their strong inducibility and low basal expression, offering superior control over recombinant protein production compared with constitutive promoters such as GAP [17, 21, 37]. Although gene copy number was inferred empirically rather than quantitatively determined, this approach has been widely adopted in *P. pastoris* expression studies and provides a practical strategy for enriching high-expression clones [23, 24].

Under optimized shake-flask induction conditions, the recombinant strain achieved an rHA1 concentration of approximately 0.375 g/L in the culture supernatant. This yield is comparable to or exceeds those reported for other viral antigens expressed in *P. pastoris*, including influenza HA variants and SARS-CoV-2 receptor-binding domain proteins produced under non-fermenter conditions [38–42]. These findings indicate that the constructed expression platform performs efficiently at the microbial production level.

Efficient downstream processing is critical for the feasibility of microbial protein manufacturing. In this study, rHA1 was readily purified from the culture supernatant using Ni-NTA affinity chromatography, yielding protein with a purity exceeding 95%, consistent with previous reports employing secretion-based purification strategies in *P. pastoris* [43–45]. PNGase F digestion confirmed the presence of N-linked glycosylation, a modification known to contribute to correct folding and stability of HA proteins [33, 34]. Although detailed glycan profiling was beyond the scope of this study, the observed molecular weight shift and electrophoretic consistency suggest appropriate eukaryotic processing compatible with downstream applications.

Although the present study primarily focused on microbial production technologies, limited biological validation to verify that the yeast-expressed rHA1 retained fundamental functional properties. Mouse immunization induced rHA1-specific IgG responses and hemagglutination-inhibiting antibodies, indicating preservation of conformational epitopes required for HA function. These observations are consistent with previous studies of recombinant HA proteins expressed in eukaryotic systems [46, 47]. The in vivo assays therefore served as supportive evidence of antigen integrity rather than a comprehensive evaluation of vaccine efficacy.

Cytokine expression and lymphocyte proliferation assays further indicated that the recombinant HA1 protein was biologically active. These analyses were not intended to dissect immune mechanisms but to complement biochemical and serological data confirming antigen functionality, a validation strategy commonly adopted in microbial expression studies of viral proteins [48–50].

Several limitations warrant consideration. Gene copy number was not quantitatively determined, and future studies using qPCR or Southern blot analysis may provide deeper insights into expression-yield relationships. Additionally, production was assessed under shake-flask conditions; further optimization in controlled bioreactor systems may significantly enhance productivity and consistency, as reported in previous *P. pastoris* fermentation studies [51, 52]. Finally, advanced structural and glycosylation analyses were beyond the scope of the present work but may be pursued depending on the intended application of the recombinant protein.

## Conclusion

In conclusion, this study establishes a highly efficient *P. pastoris*-based platform for the production of recombinant H1N1 HA1 protein, achieving gram-per-liter–level yields while preserving protein quality and functional integrity. Through codon optimization, AOX1-driven expression, multi-copy genomic integration, and secretion-based production, we demonstrate that *P. pastoris* can overcome key bottlenecks associated with the expression of complex viral glycoproteins.

The resulting rHA1 protein exhibits high purity, appropriate post-translational modification, and retained biological functionality, underscoring the potential of *P. pastoris* as a scalable and cost-effective microbial cell factory for recombinant influenza antigen production. These findings provide a strong technical foundation for the rapid manufacture of HA-based antigens and support further development toward industrial and translational applications.

## Data Availability

All data generated or analyzed during this study are included in this published article. Additional datasets supporting the conclusions are available from the corresponding author upon reasonable request.
